# The protocadherin 11X/Y (*PCDH11X/Y*) gene pair as determinant of cerebral asymmetry in modern *Homo sapiens*

**DOI:** 10.1111/nyas.12042

**Published:** 2013-04-18

**Authors:** Thomas H Priddle, Timothy J Crow

**Affiliations:** Department of Psychiatry, University of OxfordOxford, United Kingdom

**Keywords:** human evolution, language, Xq21.3/Yp11.2, PCDH11X/Y, neurodevelopment

## Abstract

Annett's right-shift theory proposes that human cerebral dominance (the functional and anatomical asymmetry or torque along the antero-posterior axis) and handedness are determined by a single “right-shift” gene. Familial transmission of handedness and specific deviations of cerebral dominance in sex chromosome aneuploidies implicate a locus within an X–Y homologous region of the sex chromosomes. The Xq21.3/Yp11.2 human-specific region of homology includes the protocadherin 11X/Y (*PCDH11X/Y*) gene pair, which encode cell adhesion molecules subject to accelerated evolution following the separation of the human and chimpanzee lineages six million years ago. *PCDH11X* and *PCDH11Y*, differentially regulated by retinoic acid, are highly expressed in the ventricular zone, subplate, and cortical plate of the developing cerebral cortex. Both proteins interact with β-catenin, a protein that plays a role in determining axis formation and regulating cortical size. In this way, the *PCDH11X/Y* gene pair determines cerebral asymmetry by initiating the right shift in *Homo sapiens*.

## Introduction

In his “‘Recapitulation and Conclusion,” Darwin wrote, “In the distant future I see open fields for far more important researches. Psychology will be based on a new foundation, that of the necessary acquirement of each mental power and capacity by gradation. Light will be thrown on the origin of man and his history” emphasizing the gradual nature of the transition that he envisaged between species.^1^ In this he was challenged by Thomas Huxley who wrote on publication of *On the Origin of Species* “I hope that you have not loaded yourself with an un-necessary difficulty in adopting *Natura non facit saltum* so unreservedly.” Not long after this, Huxley was engaged in public debate with Darwin's adversary Richard Owen on the question of what characterized the human brain.^2^ Owen had proposed it was the hippocampus minor. Huxley responded that the hippocampus minor was a trivial consequence of the structure of the lateral ventricle, reinforcing his argument with a comparative account of the relationship between these structures in the great apes.

Neither Huxley nor Owen appears to have been struck by the potential relevance to their debate of the observation of Broca that the seat of language was located in the frontal lobe, and lateralized to the left. Nor did Paul Broca initially perceive the implications of his observation for the differences between species of great ape. But in 1877, in a festschrift for his colleague Armand de Fleury, he stated, “Man is, of all the animals, the one whose brain …is most asymmetrical…also possesses most acquired faculties. …the faculty of language distinguishes us most clearly from the animals.”^3^ In proposing a specific role for asymmetry, Broca was preceded by Pierre Gratiolet, who described how the gyri on the left side of the frontal lobe develop earlier than those on the right side, and those of the occipital lobes develop on the right before the left.^4^ Although he did not use the term, Gratiolet can be regarded as the originator of the concept of a *cerebral torque*, the notion that brain asymmetries are not simply a left–right difference, but a gradient that crosses the antero-posterior (A–P) axis, a phenomenon rediscovered over 125 years later.^5^

## The right shift

The thesis that asymmetry, including its functional as well as its anatomical aspect, is the defining characteristic of the human brain was defended in the second part of the 20th century, particularly by Marian Annett.^6^,^7^ Annett's theory is based upon three rules:

1. A single gene for left-hemisphere speech coincidentally biases handedness to the right.
2. Directional asymmetry is specific to humans.
3. The dimension of asymmetry influences critical aspects of human cognitive ability.

Concerning the first rule, there is general agreement that a single gene can, indeed, account for the inheritance of handedness within families,^6^,^8^,^9^ although some researchers have nevertheless argued for multiple genes with an additive effect, as reviewed by Corballis.^10^

The second rule attracts less agreement. In neuroanatomical studies of the chimpanzee brain, there are claims for^11^,^12^ and against^13^,^14^ the presence of systematic asymmetries. Concerning nonhuman primate handedness, meta-analyses suggest that, although individual nonhuman primates may be right- or left-handed, on a population basis there is no directional asymmetry.^15^,^16^ Furthermore, handedness in nonhuman primates has been shown to be task and tool specific.^17^,^18^ There is evidence that population-level right-handedness was present in Neanderthals, and possibly other hominins,^19^ and therefore entered the genus *Homo* after the divergence of the chimpanzee lineage, within the last 6 million years,^20^,^21^ although the question of the language capability of Neanderthals is debated.^22^,^23^

**Figure 1:**
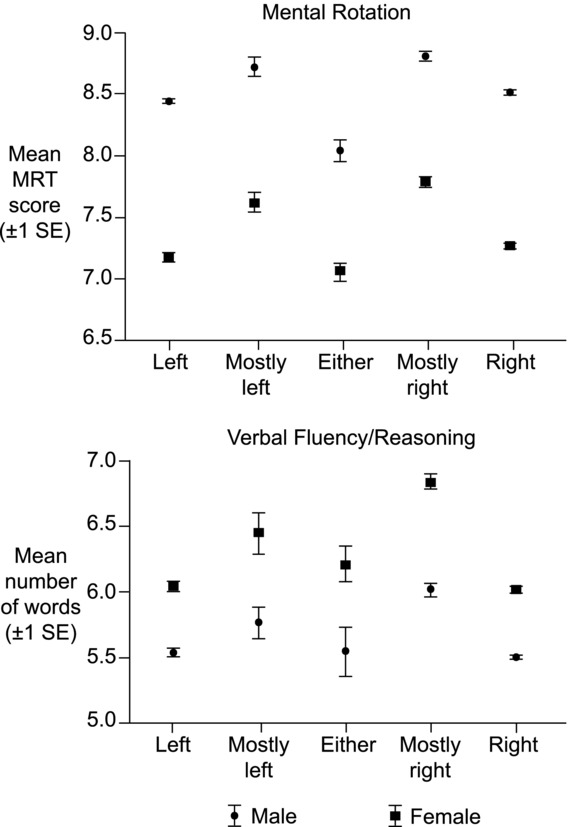
The BBC Internet survey. Mental rotation (top) and verbal fluency/reasoning (bottom) test scores, as functions of writing hand preference. Note the better performance by males for the mental rotation task and by females for the verbal fluency/reasoning task. Data adapted from Ref. 26.

Several studies have now addressed the third rule and demonstrated a relationship between the degree of handedness and aspects of cognitive ability.^24^–^29^ In 12,770 children in the UK National Child Development Study (UKNCDS), relative hand skill was found to predict verbal ability, nonverbal ability, mathematical skill, and reading skill; those at the extremes of handedness were modestly impaired relative to those with more moderate handedness, but those who were most impaired were those individuals close to ambidexterity, referred to as the *point of hemispheric indecision*. Females demonstrated greater mean verbal ability and mean mathematical ability was greater in males, but the form of the relationship with hand skill was the same in both sexes.^24^ A similar relationship, between hand preference and verbal and nonverbal ability was observed in the BBC Internet sample of 250,100 adults surveyed in relation to a television program on sex differences,^26^ that found an M-shaped relationship (Fig. 1) between self-rated degrees of handedness and ability, closely similar in form in the two sexes but with an advantage to females for verbal and males for nonverbal or spatial ability. The Avon Longitudinal Study of Parents and Children (ALSPAC) noted only modest developmental deficits in left-handed children (between 41 months and 14 years of age) but a much larger disadvantage to mixed-handers.^27^ Interestingly, a national survey of development in four to five year olds found left- and particularly mixed-handers performed significantly worse than right-handers on tests of general cognitive ability (GCA) and receptive English skills, but were unimpaired on the Peabody Picture Vocabulary test and tests of expressive English.^28^ Reanalysis of the UKNCDS data with multiple regression confirmed that increased laterality in either direction improved cognitive ability.^25^ Some inconsistencies may result from the ways in which handedness can be categorized. For instance, when Nicholls *et al*. recorded hand preference there was a barely significant effect in relation to GCA, but when hand performance was measured there was a distinct GCA advantage for right-handers with deficits for extreme left- and right-handedness.^29^ However, no disadvantage to mixed-handers was detectable in this study. While it has been suggested that some left-handers may be so-called pathological left-handers, individuals whose choice of hand was forced as a result of an early left brain insult,^30^,^31^ no evidence of this was detected in the developmental cohort of Johnston *et al*.^28^

Annett's rules converge to predict a biallelic right-shift (RS) gene. One allele (RS^+^) determines left-cerebral language dominance and, coincidentally, biases handedness toward the right, and the other allele (RS^−^) leaves both asymmetries to chance. Approximately 30% of the general population are predicted to be homozygous carriers of the RS^+^ allele (RS^+/+^), for whom right-cerebral language development is severely curtailed; less than 1% are predicted to write with the left hand. Heterozygotes (RS^+/−^) are predicted to make up approximately 50% of the general population and 8% of them will be left-handed. The remaining 20% or so are homozygous carriers of the RS^−^ allele (RS^−/−^) and will develop hand preference at random (50% left, 50% right) with many developing mixed-handedness. However, Annett suggests that social pressure brings the figure for left-handed writing in this group down to 33%. Together, these rates of hand preference approach those observed, i.e., 3–4% pure left-handers, 25–33% mixed-handedness, and 60–70% pure right-handers.^7^

## The location of the right shift

The aforementioned sex differences, together with observations that females are more strongly right-handed,^32^ males have a greater tendency toward left-handedness,^33^ and that the torque is anatomically more pronounced in males,^34^ implicate a sex chromosomal locus for the right shift. Further evidence of an X/Y homologous sex chromosomal locus for cerebral asymmetry comes from cases of sex chromosomal aneuploidies; individuals with Turner's syndrome, who have only one X chromosome, or individuals with an extra X (triple X and Klinefelter's [XXY] syndromes) or an extra Y (XYY syndrome). In these syndromes there are reciprocal deficits that can be attributed to the two hemispheres. Individuals with Turner's syndrome have deficits in spatial ability usually attributed to the right hemisphere.^35^–^37^ Patients with an extra X,^38^–^40^ or, importantly, an extra Y,^41^,^43^ have delays in language acquisition attributable to the left hemisphere. These neuropsychological deficits are supported by anatomical deviations along the A–P axis.^44^ Furthermore, an increase in mixed- or left-handedness is seen in patients with Klinefelter's syndrome,^45^,^46^ and Turner's patients present reduced right hand dominance.^47^ Both sexes demonstrate similar deficits, despite the differing hormonal profiles of males and females with these conditions.^38^,^43^ Thus, not only are these deficits genetic in origin, but the gene is located in a region of X/Y homology that escapes X inactivation.^48^

If the locus for cerebral asymmetry is present on the sex chromosomes, then predictions can be made concerning the transmission of handedness from a father to his offspring. An allele on the X chromosome would be passed only to daughters. Likewise, an allele on the Y chromosome would be passed only to sons. Handedness would more often than not be passed to siblings of the same sex. This prediction was confirmed in a large investigation of 14,500 sibling pairs, with the study providing support for: “complete rather than partial X–Y linkage, that is, an homologous gene in the sex-specific, rather than in the pseudo-autosomal, region of the sex chromosomes.”^49^

**Figure 2:**
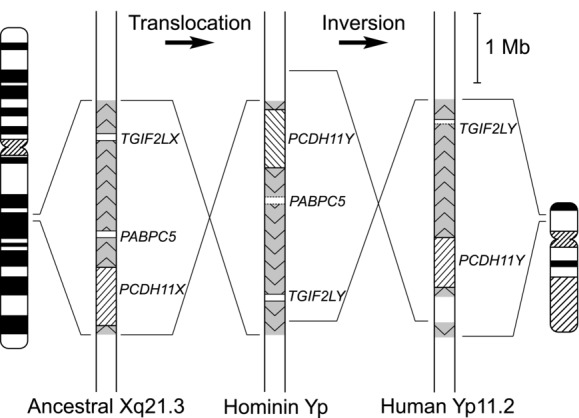
The Xq21.3/Yp11.2 reduplicative translocation. The creation of the human Yp11.2 region began 6 million years ago when the Xq21.3 block was duplicated onto the hominin Y chromosome. The Yp11.2 block inverted at a later date (presently unknown), *PABPC5* was deleted, and *TGIF2LY* was truncated.

One such locus, the Xq21.3–Yp11.2 homology region, stands out in recent evolution. The Yp11.2 region was duplicated from the long arm of the X chromosome (Xq21.3) onto the Y chromosome short arm (shown in Fig. 2) around 6 million years ago, i.e., the time of separation of the hominin and chimpanzee lineages, and the block was subsequently split by a currently undated paracentric inversion.^21^ This X-transposed region (XTR) is not present in chimpanzees or other mammals.^50^

At the time of the translocation there were three genes within the region. One, a poly(A)-binding gene (*PABPC5*), has subsequently been deleted from the Y.^51^ A second gene (*TGIF2LX/Y*) remains on both chromosomes, but the Y-linked copy has been subject to a single base pair deletion that truncates the protein.^52^ The remaining gene pair, protocadherin 11X/Y (*PCDH11X/Y*), is a member of a large family of transmembrane cell adhesion molecules expressed predominantly in the brain, that forms the largest cadherin superfamily.^53^,^54^ Classical cadherins are involved in morphogenesis through calcium-dependent homophilic cell adhesion mediated by a conserved motif in the first cadherin repeat (EC1) of the ectodomain.^55^ Because this motif is absent from EC1 of protocadherins (PCDHs), they are thought to play a role in the specificity of cell–cell connections rather than contribute to their strength.^56^ PCDHs are classified into α, β, and γ subfamilies based on their clustered genetic organization.^57^ An additional nonclustered group, termed δ-PCDHs, can be further subdivided, based on the number of ECs and features of their cytodomains, into δ1- (the group containing PCDH11X/Y) and δ2-PCDHs.^58^,^59^ δ-PCDHs are involved in many critical aspects of neurodevelopment.^58^,^60^ Much of our understanding of the mechanism of extracellular PCDH binding comes from work on the clustered PCDHs.^61^ *Trans* interactions (across the cell junction) are strictly homophilic and require less calcium than classical cadherins. In contrast, *cis* interactions (on the same cell surface) show no isoform specificity and can be mediated by either covalent disulphide bonding or noncovalent bonds. A tetramer of *cis* interacting units binds homophilically to an equivalent tetramer in *trans*, via EC2 and EC3. These features, together with its genomic location, make PCDH11X/Y the prime candidate for the right shift and therefore for determining cerebral asymmetry in *H. sapiens*.^62^,^63^

## Protocadherin 11X/Y

**Figure 3:**
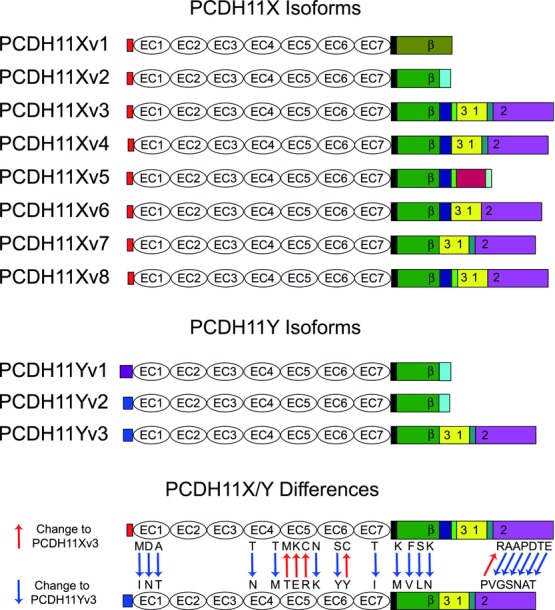
PCDH11X/Y isoforms and sequence differences. PCDH11X (top) and PCDH11Y (middle) isoforms differ principally in their cytodomains and signal peptides, shown in color. There are several amino acid differences between PCDH11X and PCDH11Y (bottom). EC1–7, ectodomains 1–7; β, β-catenin binding site; 1–3, conserved motifs 1–3.

The basic structures of the PCDH11X/Y proteins are similar (PCDH11X and PCDH11Y are 98.1% identical); each comprises an ectodomain with 7 ECs, a short transmembrane region, and a variable length cytodomain that defines the isoforms.^58^,^64^–^66^ The isoforms are derived from a pool of up to 28 exons that encodes a theoretical maximum of 360 variants,^66^ with at least eight X and three Y-linked isoforms experimentally validated (Fig. 3).^58^,^60^,^66^,^67^

Following the translocation, *PCDH11X/Y* has undergone accelerated evolution in the hominin lineage.^21^ The outstanding questions are which changes are *Homo sapiens* specific, and at what point during the evolution of hominins may they have contributed to the development of handedness? The sequence of the Neanderthal sex chromosomes, including Xq21.3/Yp11, is presently incomplete.^68^ We retrieved the following data from the Neanderthal Genome Browser (http://projects.ensembl.org/neandertal/). Most samples sequenced to date are female, so there are some data for PCDH11X, but few for PCDH11Y. The PCDH11Y ectodomain has accumulated eight changes since the common great ape/hominin precursor, for which Neanderthal sequence is available for only one, and it reflects the human sequence. Since the common precursor there have been 10 changes in the PCDH11Y cytodomain,^21^ and a further 39 amino acids have been deleted (although the sequence remains in-frame and the gene is expressed in the brain). Information on Neanderthal/human differences in the critical region of the Y chromosome is therefore limited. In the PCDH11X ectodomain, there have been four coding changes since the chimpanzee/hominin divergence. Three of these sites have been sequenced for the Neanderthal genome project and reflect the human sequence. These sites are located in EC5, and 3D homology modeling predicts that they are mapped closely to one another in space.^69^ One change, Cys517, is located on the surface of EC5, unpaired to any other cysteine residue and free to form a disulphide bond. A human-specific cysteine (Cys680) is introduced between EC6 and EC7. Disulphide bonds formed between ectodomains could stabilise multimers of PCDH11X at the cell surface, a mechanism previously described for the *Xenopus* δ2-family member paraxial protocadherin,^70^ and γ-Pcdh-A3 tetramers.^61^ In the longest PCDH11X cytodomain, a single change is present in both humans and Neanderthals. The PCDH11X EC5 and cytodomain changes cannot therefore be implicated in any characteristic of modern *H. sapiens*. They may have appeared recently and distinguish modern *H. sapiens* and *H. neanderthalensis* from earlier hominins, or they may have appeared earlier facilitating, for example, an increase in brain size (see below) in the genus *Homo*. The undated paracentric inversion may also be considered a candidate for pre-*H. neanderthalensis / H. sapiens* events such as upright walking. Nevertheless, until a definitive Neanderthal sequence is produced, PCDH11X Cys680 and seventeen of the PCDH11Y changes remain candidates for human-specific characteristics.

The cytodomain of PCDH11X/Y has been shown to interact with β-catenin,^67^ and induces the Wnt signaling pathway in cultured prostate cancer cells.^71^ Longer variants also interact with protein phosphatase 1α (PP1α) via the δ1-PCDH-specific conserved motif 3 (CM3).^59^

Retinoic acid influences the expression of PCDH11X/Y mRNA in opposite directions, downregulating *PCDH11X* and upregulating *PCDH11Y*.^65^ PCDH11X/Y mRNA is expressed in many regions of the fetal and adult human brain, including frontal, temporal, and occipital cortex, corpus callosum, amygdala, hippocampus, caudate nucleus, thalamus, substantia nigra, and cerebellum.^65^,^66^,^72^ Real-time PCR has demonstrated twice as much PCDH11X mRNA in adult female temporal lobes than in males.^73^ A longitudinal study of the prefrontal cortex using microarrays concluded that levels of PCDH11X/Y are highest in male neonates, decrease through childhood, and are lowest in adults of both sexes.^74^ A similar expression profile is observed for PCDH11Y (only) across multiple brain regions.^75^

In a recent immunohistochemical survey of fetal (12–34 weeks postconception) and adult human brains, PCDH11X/Y expression was detected in developing neurons as they migrated from the ventricular zone (VZ), through the subplate and into the cortical plate (CP).^76^ Expression was detected in interneurons of the developing cerebral cortex, thalamus, caudate, putamen, and the ganglionic eminences from which many of these cells originate, the hippocampal formation, brainstem, Purkinje cells, and deep nuclei of the cerebellum. These expression patterns were maintained in the mature structures of the adult brain. The study found no gender differences in expression but could not distinguish the highly homologous PCDH11X and PCDH11Y. To overcome this difficulty we are investigating PCDH11X/Y expression *in situ* using locked nucleic acid probes, a technology that increases probe specificity when hybridizing to sequences that differ by a few bases.^77^

Several exonic single nucleotide polymorphisms (SNPs) have been identified in the *PCDH11X/Y* sequence.^72^,^78^,^79^ The majority are synonymous (silent), but some are nonsynonymous (coding) changes. Two within the PCDH11Y cytodomain are always found together and define the ancestral allele of *PCDH11Y*.^80^ The canonical *PCDH11Y* sequence, without these changes, is the derived allele.^80^ One of these changes is located within the β-catenin binding site that, in the ancestral allele, has the same sequence as *PCDH11X*. The functional significance of this difference is as yet unknown. Nevertheless, it is reasonable to assume that the interaction with β-catenin differs between the sexes and between males expressing the different alleles. No association of the alleles to psychosis has been observed.^72^,^78^ An unrelated, intronic SNP was associated with late onset Alzheimer's disease in women,^81^ but several later studies have failed to replicate this association.^82^–^85^

Independent intragenic deletions in both Xq21.3 and Yp11.2 involving *PCDH11X* and *PCDH11Y* have been reported in a case of a male child with severe language delay.^86^ The *PCDH11Y* deletion was a *de novo* occurrence, not present in the father, while the *PCDH11X* deletion was inherited from the (phenotypically normal) mother. The authors postulate that the deletions interfere with normal splicing, altering gene expression to disrupt the development of language. In another study two brothers with intellectual disability were identified with a 182 Kb duplication within intron 2 of *PCDH11X*, although their mildly affected sister was found not to carry the duplication.^87^ One interpretation of these findings is that an interruption of *PCDH11X* is less well tolerated in males than in females, a possible explanation of the male propensity to autism and attention deficit hyperactivity disorder.^88^

## Cerebral asymmetry

The formation of the left–right (L–R) axis is preceded by the specification of the dorso–ventral (D–V) and A–P axes.^89^,^90^ Retinoic acid is crucial for the establishment of both the neural D–V and the A–P axes forming a gradient along each.^91^ β-Catenin is required for D–V axis formation in a number of species including zebrafish,^92^ *Xenopus*,^93^ and mice^94^ as a component of the canonical Wnt signaling pathway. Wnt signaling is also crucial for the establishment of the A–P axis,^95^ and involved in L–R axis determination of the body^96^ and brain.^97^

Wnt binds to frizzled-type receptors and activates disheveled, which downregulates the destruction complex comprising axin, adenomatous polyposis coli (APC), casein kinase 1 (CK1), and glycogen synthase kinase 3β (GSK-3β) that would normally phosphorylate β-catenin, marking it for degradation by the multiprotein ubiquitin–proteasome complex.^98^ Unphosphorylated β-catenin accumulates within the cytoplasm and then enters the nucleus and coactivates transcription with T cell (TCF) and lymphoid enhancement factors (LEF). Conversely, the inhibition of PP1α stimulates the degradation of β-catenin by increasing the phosphorylation of Axin by CK1, which in turn enhances GSK-3β binding to β-catenin.^99^ The δ1-PCDH, Pcdh7c, inhibits PP1α via the CM3 motif^100^ that is also present in PCDH11X/Y.^59^

PCDH11X/Y's interactions with β-catenin^67^,^71^ and PP1α^59^ have the potential to mediate a feedback loop regulating levels of cytoplasmic β-catenin, and thereby influence the formation of the D–V, A–P, and L–R axes. Whether PCDH11X/Y can bind both β-catenin and PP1α at the same time should be investigated further.

The proto-map hypothesis proposes that an increase in divisions of cells within the radial columnar units of a developing human brain leads to an enlarged cerebral cortex relative to other primates.^101^,^102^ Cell divisions within the VZ proceed symmetrically during the first phase of cortical development; each cell divides in two and each of these daughter cells divide in two and so on, leading to an exponential increase in the number of cells. As development proceeds, these cells begin to divide asymmetrically; one daughter cell stops dividing and leaves the VZ by traveling up the process of the radial glial cells into the CP, while the other remains within the VZ, continuing to divide. In humans, this occurs around Carnegie stage 21 (CS21), approximately the seventh week of gestation.^103^

β-Catenin is highly expressed in the neuronal precursors of the VZ and thought to influence the decision of these cells to reenter the cell cycle rather than to differentiate and migrate into the CP.^104^ Transgenic mice expressing an engineered form of β-catenin lacking the normal phosphorylation sites exhibit enlarged brains with an increased surface area, and folds resembling sulci and gyri, normally absent in the mouse cerebral cortex.^104^, ^105^ β-Catenin signaling negatively regulates the production of intermediate progenitor cells in the subventricular zone (SVZ),^106^ a region that is hypothesized to facilitate the expansion of the cortex over evolutionary time.^107^,^108^

**Figure 4:**
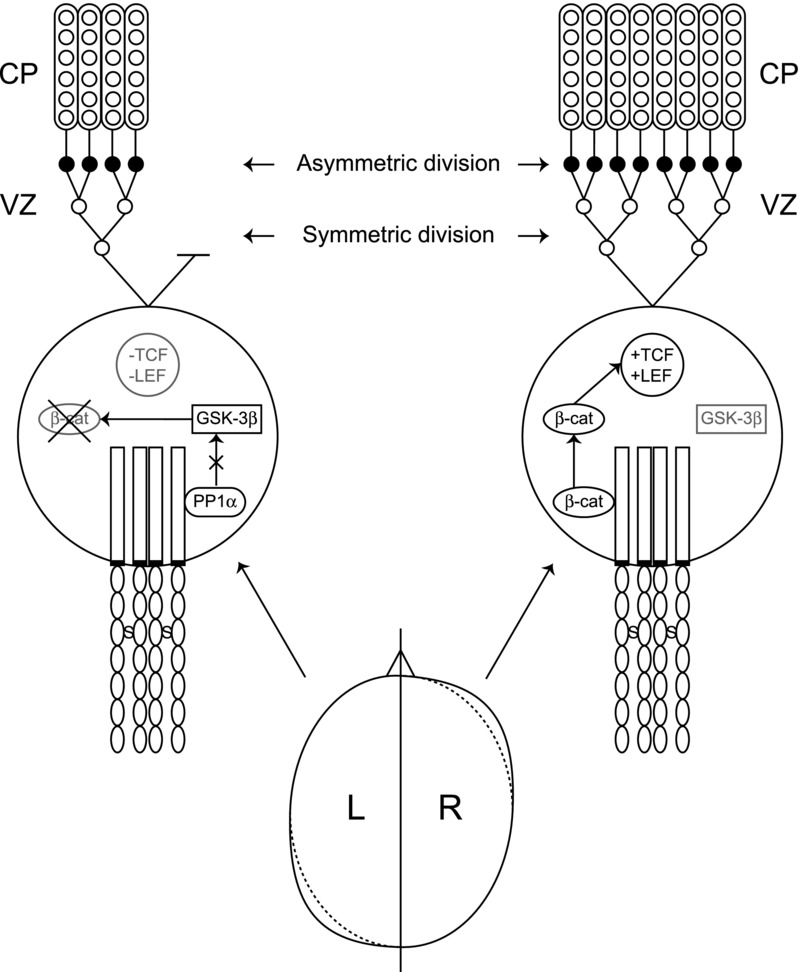
Proposed mechanism by which PCDH11X/Y alters cortical surface area along the A–P axis. PCDH11X/Y tetramers, stabilized by disulphide bonds (S), are shown at the surface of neuronal precursors within the ventricular zone (VZ). In the left-frontal region (and right-occipital region, omitted for clarity) PCDH11X/Y inhibits protein phosphatase 1α (PP1α). This stimulates the degradation of β-catenin (β-cat) by enhancing the activity of glycogen synthase kinase 3β (GSK-3β). β-Catenin can no longer coactivate transcription with T cell (TCF) and lymphoid enhancement factors (LEF) and the cell stops dividing symmetrically and begins asymmetric division preventing the pool of neuronal precursors from expanding. In the right-frontal region (and left-occipital region, omitted for clarity) PCDH11X/Y protects β-catenin from degradation by GSK-3β. β-Catenin can coactivate transcription with TCF and LEF, and the cell continues dividing symmetrically, thus increasing the pool of neuronal precursors before the phase of asymmetric division, leading to an increase in the number of cell columns within the cortical plate (CP). See the text for further information.

We propose that, prior to CS21, PCDH11X/Y accumulates along the A–P and L–R axes in response to retinoic acid. PCDH11X/Y tetramers form on the surface of the neuronal precursors within the VZ, facilitated by the human-specific disulphide bonding of PCDH11X. In the right-frontal and left-occipital regions, PCDH11X/Y promotes the accumulation of β-catenin in the neuronal precursors of the VZ, promoting additional rounds of symmetric cell division within the neuronal precursor pool. This increase in the precursor pool before the phase of asymmetrical division when cells migrate into the CP increases the number of cell columns, thus increasing cortical surface area. In the left-frontal and right-occipital regions, PCDH11X/Y inhibits PP1α, which stimulates the breakdown of β-catenin, triggering the switch from symmetrical division to asymmetrical division earlier. The precursor pool does not expand as much, fewer cell columns are generated, and the resulting cortical surface area is reduced relative to the right-frontal and left-occipital regions (Fig. 4).

The development of the torque is likely to be based upon differential rates of cortical expansion, which when measured as the intrinsic curvature of the cortex have been shown to differ significantly between humans and chimpanzees.^109^ Two studies indicate that cortical development in man is under epigenetic control: (1) cortical surface area and thickness are influenced by paternal and maternal age at the birth of the child, acting in opposing directions;^110^ and (2) handedness in the child is more likely to be to the left if the mother is aged less than 20 years at the time of birth, and to be to the right if the father is aged less than 20.^111^ Such effects may be due to variations in an epigenetic signal arising from meiotic silencing of unsynapsed chromatin (MSUC), the mechanism whereby only genes that are paired with homologous sequences are transcriptionally active during the later stages of meiosis, a state that persists in the resulting gamete.^112^ Therefore, in male meiosis most genes on the X and Y chromosomes are transcriptionally silenced within a specialized compartment, the XY body.^113^ However, the *PCDH11X/Y* gene pair is thought to be protected from MSUC. The physical process of pairing, particularly in the case of the Xq21.3/Yp11.2 region of homology, is likely to be variable and may therefore account for the significant variations in cerebral asymmetry.

## Conclusions

The human-specific nature of cerebral dominance and the evolutionary history of *PCDH11X/Y* make this gene pair the leading candidate for the right-shift factor. The sequence changes in the PCDH11X ectodomain occurring in the latest step in human evolution are postulated to have introduced PCDH11X/Y tetramers that interact with β-catenin to effect a deviation along the A–P axis present in females and accentuated in males. Chromosomal rearrangements have been proposed to play a role in speciation.^114^,^115^ If one considers the presence of new sequences such as *PCDH11Y* on the Y chromosome as a speciation event, it is apparent that the pattern of gene expression is both subject to sexual selection at the time of the event, and incorporates the features of the new species into the epigenetic message generated in male meiosis.^116^ Hence, critical information on the nature of the species will be epigenetically transferred to the embryo. In this manner, the next generation may receive not only the message that defines the species' characteristic, the cerebral asymmetry that in the case of *H. sapiens* yields the capacity for language, but also its variability between individuals.

## Conflicts of interest

The authors declare no conflicts of interest.

## References

[b1] Darwin C (1859). On the Origin of Species by Means of Natural Selection: Or, the Preservation of Favoured Races in the Struggle for Life.

[b2] Owen CM, Howard A, Binder DK (2009). Hippocampus minor, calcar avis, and the Huxley-Owen debate. Neurosurgery.

[b3] Broca P (1877). Rapport sur un memoire de M. Armand de Fleury intitulé: de l'inegalité dynamique des deux hemisphères cerébraux. Bulletins de l’Academie de Medicine.

[b4] Gratiolet P (1839). Anatomie Compare du Systeme Nerveux, Considere Dans ses Rapports avec L'intelligence.

[b5] Yakovlev PI, Rakic P (1966). Patterns of decussation of bulbar pyramids and distribution of pyramidal tracts on two sides of the spinal cord. Trans. Am. Neurol. Assoc.

[b6] Annett M (1985). Left, Right, Hand and Brain: The Right Shift Theory.

[b7] Annett M (2002). Handedness and Brain Asymmetry: The Right Shift Theory.

[b8] McManus IC (1985). Handedness, language dominance and aphasia: a genetic model. Psychol. Med. Monogr. Suppl.

[b9] McKeever WF (2000). A new family handedness sample with findings consistent with X-linked transmission. Br. J. Psychol.

[b10] Corballis MC (2009). The evolution and genetics of cerebral asymmetry. Philos. Trans. R. Soc. Lond. B. Biol. Sci.

[b11] Spocter MA (2010). Wernicke's area homologue in chimpanzees (Pan troglodytes) and its relation to the appearance of modern human language. Proc. R. Soc. Lond. B. Biol. Sci.

[b12] Hopkins WD, Russell JL, Cantalupo C (2007). Neuroanatomical correlates of handedness for tool use in chimpanzees (Pan troglodytes) implication for theories on the evolution of language. Psychol. Sci.

[b13] Chance S (2012). Hemispheric asymmetry in the fusiform gyrus distinguishes Homo sapiens from chimpanzees. Brain Struct. Funct.

[b14] Schenker NM (2010). Broca's area homologue in chimpanzees (Pan troglodytes): probabilistic mapping, asymmetry, and comparison to humans. Cereb. Cortex.

[b15] McGrew WC, Marchant LF (1997). On the other hand: current issues in and meta-analysis of the behavioral laterality of hand function in nonhuman primates. Am. J. Phys. Anthropol.

[b16] Palmer AR (2002). Chimpanzee right-handedness reconsidered: evaluating the evidence with funnel plots. Am. J. Phys. Anthropol.

[b17] Lonsdorf EV, Hopkins WD (2005). Wild chimpanzees show population-level handedness for tool use. Proc. Natl. Acad. Sci. US A.

[b18] Marchant L, McGrew W (2007). Ant fishing by wild chimpanzees is not lateralised. Primates.

[b19] Uomini NT (2009). The prehistory of handedness: archaeological data and comparative ethology. J. Hum. Evol.

[b20] Chen F-C, Li W-H (2001). Genomic divergences between humans and other hominoids and the effective population size of the common ancestor of humans and chimpanzees. Am. J. Hum. Genet.

[b21] Williams NA (2006). Accelerated evolution of Protocadherin11X/Y: a candidate gene-pair for cerebral asymmetry and language. Am. J. Med. Genet. B.

[b22] Frayer DW (2010). Right handed Neandertals: vindija and beyond. J. Anthropol. Sci.

[b23] Benítez-Burraco A, Longa VM (2012). Right-handedness, lateralization and language in Neanderthals: a comment on Frayer *et al*. (2010). J. Anthropol. Sci.

[b24] Crow TJ (1998). Relative hand skill predicts academic ability: global deficits at the point of hemispheric indecision. Neuropsychologia.

[b25] Nettle D (2003). Hand laterality and cognitive ability: a multiple regression approach. Brain Cogn.

[b26] Peters M, Reimers S, Manning JT (2006). Hand preference for writing and associations with selected demographic and behavioral variables in 255,100 subjects: the BBC internet study. Brain Cogn.

[b27] Gregg P, Janke K, Propper C (2008). Handedness and Child Development. Working paper 08/198 of the Centre for Market and Public Organisation.

[b28] Johnston D (2009). Nature's experiment? Handedness and early childhood development. Demography.

[b29] Nicholls MER (2010). The relationship between hand preference, hand performance, and general cognitive ability. J. Int. Neuropsychol. Soc.

[b30] Bishop DVM (1984). Using non-preferred hand skill to investigate pathological left-handedness in an unselected population. Dev. Med. Child Neurol.

[b31] Gillberg C, Waldenström E, Rasmussen P (1984). Handedness in Swedish 10-year-olds. Some background and associated factors. J. Child Psychol. Psychiat.

[b32] Tan U, Tan M (1997). The mixture distribution of left minus right hand skill in men and women. Int. J. Neurosci.

[b33] Papadatou-Pastou M (2008). Sex differences in left-handedness: a meta-analysis of 144 studies. Psychol. Bull.

[b34] Barrick TR (2005). Automatic analysis of cerebral asymmetry: an exploratory study of the relationship between brain torque and planum temporale asymmetry. Neuroimage.

[b35] Rovet JF (1993). The psychoeducational characteristics of children with Turner syndrome. J. Learn. Disabil.

[b36] Ross JL (2002). Persistent cognitive deficits in adult women with Turner syndrome. Neurology.

[b37] Kesler SR (2004). Functional neuroanatomy of spatial orientation processing in Turner syndrome. Cereb. Cortex.

[b38] Netley C, Rovet J (1982). Verbal deficits in children with 47, XXY and 47, XXX karyotypes: a descriptive and experimental study. Brain Lang.

[b39] Boone KB (2001). Neuropsychological profiles of adults with Klinefelter syndrome. J. Int. Neuropsychol. Soc.

[b40] Rovet J, Netley C (1983). The triple X chromosome syndrome in childhood: recent empirical findings. Child Dev.

[b41] Bender B (1983). Speech and language development in 41 children with sex chromosome anomalies. Pediatrics.

[b42] Walzer S, Bashir AS, Silbert AR (1990). Cognitive and behavioral factors in the learning disabilities of 47,XXY and 47,XYY boys. Birth Defects Orig. Artic. Ser.

[b43] Geerts M, Steyaert J, Fryns JP (2003). The XYY syndrome: a follow-up study on 38 boys. Genet. Couns.

[b44] Rezaie R (2008). The influence of sex chromosome aneuploidy on brain asymmetry. Am. J. Med. Genet. B.

[b45] Netley C, Rovet J (1982). Handedness in 47,XXY males. Lancet.

[b46] Geschwind DH (1998). Klinefelter's syndrome as a model of anomalous cerebral laterality: testing gene dosage in the X chromosome pseudoautosomal region using a DNA microarray. Dev. Genet.

[b47] Bender BG, Linden MG, Robinson A (1993). Neuropsychological impairment in 42 adolescents with sex chromosome abnormalities. Am. J. Med. Genet.

[b48] Crow TJ (1994). The case for an X–Y homologous determinant of cerebral asymmetry. Cytogenet. Cell Genet.

[b49] Corballis MC (1996). Location of the handedness gene on the X and Y chromosomes. Am. J. Med. Genet.

[b50] Hughes JF (2010). Chimpanzee and human Y chromosomes are remarkably divergent in structure and gene content. Nature.

[b51] Blanco P (2001). A novel poly(A)-binding protein gene (PABPC5) maps to an X-specific subinterval in the Xq21.3/Yp11.2 homology block of the human sex chromosomes. Genomics.

[b52] Blanco-Arias P, Sargent CA, Affara NA (2002). The human-specific Yp11.2/Xq21.3 homology block encodes a potentially functional testis-specific TGIF-like retroposon. Mamm. Genome.

[b53] Frank M, Kemler R (2002). Protocadherins. Curr. Opin. Cell Biol.

[b54] Hulpiau P, van Roy F (2009). Molecular evolution of the cadherin superfamily. Int. J. Biochem. Cell Biol.

[b55] Gumbiner BM (2005). Regulation of cadherin-mediated adhesion in morphogenesis. Nat. Rev. Mol. Cell Biol.

[b56] Morishita H, Yagi T (2007). Protocadherin family: diversity, structure, and function. Curr. Opin. Cell Biol.

[b57] Wu Q, Maniatis T (1999). A striking organization of a large family of human neural cadherin-like cell adhesion genes. Cell.

[b58] Redies C, Vanhalst K, van Roy F (2005). δ-Protocadherins: unique structures and functions. Cell. Mol. Life Sci.

[b59] Vanhalst K (2005). δ-Protocadherins: a gene family expressed differentially in the mouse brain. Cell. Mol. Life Sci.

[b60] Kim S-Y (2011). Non-clustered protocadherin. Cell Adh. Migr.

[b61] Schreiner D, Weiner JA (2010). Combinatorial homophilic interaction between γ-protocadherin multimers greatly expands the molecular diversity of cell adhesion. Proc. Natl. Acad. Sci. USA.

[b62] Crow TJ (2009). Where and what is the right shift factor or cerebral dominance gene? A critique of Francks et al. (2007). Laterality.

[b63] Priddle TH, Crow TJ (2009). The protocadherin 11X/Y gene pair as a putative determinant of cerebral dominance in Homo sapiens. Future Neurol.

[b64] Yoshida K, Sugano S (1999). Identification of a novel protocadherin gene (PCDH11) on the human XY homology region in Xq21.3. Genomics.

[b65] Blanco P (2000). Conservation of PCDHX in mammals; expression of human X/Y genes predominantly in brain. Mamm. Genome.

[b66] Blanco-Arias P, Sargent CA, Affara NA (2004). Protocadherin X (PCDHX) and Y (PCDHY) genes; multiple mRNA isoforms encoding variant signal peptides and cytoplasmic domains. Mamm. Genome.

[b67] Chen M (2002). The emergence of protocadherin-PC expression during the acquisition of apoptosis-resistance by prostate cancer cells. Oncogene.

[b68] Green RE (2010). A draft sequence of the Neandertal genome. Science.

[b69] Priddle TH, Lee WH, Crow TJ, Yoshida K (2010). The protocadherin 11X/Y gene pair and the evolution of the hominin brain. Molecular and Functional Diversities of Cadherin and Protocadherin.

[b70] Chen X (2007). Structural elements necessary for oligomerization, trafficking, and cell sorting function of paraxial protocadherin. J. Biol. Chem.

[b71] Yang X (2005). A human- and male-specific protocadherin that acts through the wnt signaling pathway to induce neuroendocrine transdifferentiation of prostate cancer cells. Cancer Res.

[b72] Durand CM (2005). Expression and genetic variability of PCDH11Y, a gene specific to Homo sapiens and candidate for susceptibility to psychiatric disorders. Am. J. Med. Genet. B.

[b73] Lopes A (2006). Inactivation status of PCDH11X: sexual dimorphisms in gene expression levels in brain. Hum. Genet.

[b74] Weickert CS (2009). Transcriptome analysis of male–female differences in prefrontal cortical development. Mol. Psychiatry.

[b75] Kang HJ (2011). Spatio-temporal transcriptome of the human brain. Nature.

[b76] Priddle TH, Crow TJ (2012). Protocadherin 11X/Y a human-specific gene pair: an immunohistochemical survey of fetal and adult brains. Cereb. Cortex.

[b77] Thomsen R, Nielsen PS, Jensen TH (2005). Dramatically improved RNA in situ hybridization signals using LNA-modified probes. RNA.

[b78] Giouzeli M (2004). ProtocadherinX/Y, a candidate gene-pair for schizophrenia and schizoaffective disorder: a DHPLC investigation of genomic sequence. Am. J. Med. Genet. B.

[b79] Lopes AM, Calafell F, Amorim A (2004). Microsatellite variation and evolutionary history of PCDHX/Y gene pair within the Xq21.3/Yp11.2 hominid-specific homology block. Mol. Biol. Evol.

[b80] Giouzeli M (2002). Detection of ProtocadherinX/Y SNPs in patients with schizophrenia and other family members. Am. J. Med. Genet.

[b81] Carrasquillo MM (2009). Genetic variation in PCDH11X is associated with susceptibility to late-onset Alzheimer's disease. Nat. Genet.

[b82] Beecham GW (2010). PCDH11X variation is not associated with late-onset Alzheimer disease susceptibility. Psychiatr. Genet.

[b83] Lescai F (2010). Failure to replicate an association of rs5984894 SNP in the PCDH11X gene in a collection of 1,222 Alzheimer's disease affected patients. J. Alzheimer's Dis.

[b84] Wu Z-C (2010). Lack of association between PCDH11X genetic variation and late-onset Alzheimer's disease in a Han Chinese population. Brain Res.

[b85] Miar A (2011). Lack of association between protocadherin 11-X/Y (PCDH11X and PCDH11Y) polymorphisms and late onset Alzheimer's disease. Brain Res.

[b86] Speevak MD, Farrell SA (2011). Non-syndromic language delay in a child with disruption in the Protocadherin11X/Y gene pair. Am. J. Med. Genet. B.

[b87] Whibley AC (2010). Fine-scale survey of X chromosome copy number variants and indels underlying intellectual disability. Am. J. Hum. Genet.

[b88] Kopsida E (2009). The role of the Y chromosome in brain function. Open Neuroendocr. J.

[b89] Ramsdell AF, Yost HJ (1998). Molecular mechanisms of vertebrate left-right development. Trends Genet.

[b90] Levin M (2004). The embryonic origins of left-right asymmetry. Crit. Rev. Oral Biol. Medicine.

[b91] Maden M (2002). Retinoid signalling in the development of the central nervous system. Nat. Rev. Neurosci.

[b92] Schneider S (1996). β-catenin translocation into nuclei demarcates the dorsalizing centers in frog and fish embryos. Mech. Dev.

[b93] Larabell CA (1997). Establishment of the dorso-ventral axis in Xenopus embryos is presaged by early asymmetries in β-catenin that are modulated by the wnt signaling pathway. J. Cell Biol.

[b94] Beddington RSP, Robertson EJ (1999). Axis development and early asymmetry in mammals. Cell.

[b95] Petersen CP, Reddien PW (2009). Wnt signaling and the polarity of the primary body axis. Cell.

[b96] Rodríguez-Esteban C (2001). Wnt signaling and PKA control nodal expression and left-right determination in the chick embryo. Development.

[b97] Carl M (2007). Wnt/axin1/β-catenin signaling regulates asymmetric nodal activation, elaboration, and concordance of CNS asymmetries. Neuron.

[b98] Nelson WJ, Nusse R (2004). Convergence of wnt, β-catenin, and cadherin pathways. Science.

[b99] Luo W (2007). Protein phosphatase 1 regulates assembly and function of the β-catenin degradation complex. EMBO J.

[b100] Yoshida K (1999). BH-protocadherin-c, a member of the cadherin superfamily, interacts with protein phosphatase 1 alpha through its intracellular domain. FEBS Lett.

[b101] Rakic P (1995). A small step for the cell, a giant leap for mankind: a hypothesis of neocortical expansion during evolution. Trends Neurosci.

[b102] Rakic P (2009). Evolution of the neocortex: a perspective from developmental biology. Nat. Rev. Neurosci.

[b103] O'Rahilly R, Gardner E (1971). The timing and sequence of events in the development of the human nervous system during the embryonic period proper. Anat. Embryol.

[b104] Chenn A, Walsh CA (2002). Regulation of cerebral cortical size by control of cell cycle exit in neural precursors. Science.

[b105] Chenn A, Walsh CA (2003). Increased neuronal production, enlarged forebrains and cytoarchitectural distortions in β-catenin overexpressing transgenic mice. Cereb. Cortex.

[b106] Mutch CA (2010). Beta-catenin signaling negatively regulates intermediate progenitor population numbers in the developing cortex. PLoS ONE.

[b107] Kriegstein A, Noctor S, Martínez-Cerdeño V (2006). Patterns of neural stem and progenitor cell division may underlie evolutionary cortical expansion. Nat. Rev. Neurosci.

[b108] Martínez-Cerdeño V, Noctor SC, Kriegstein AR (2006). The role of intermediate progenitor cells in the evolutionary expansion of the cerebral cortex. Cereb. Cortex.

[b109] Ronan L (2011). Intrinsic curvature: a marker of millimeter-scale tangential cortico-cortical connectivity. Int. J. Neural Syst.

[b110] Shaw P (2012). Parental age effects on cortical morphology in offspring. Cereb. Cortex.

[b111] Leask SJ (2012).

[b112] Lee JT (2005). Sex chromosome inactivation: the importance of pairing. Curr. Biol.

[b113] Turner JMA (2007). Meiotic sex chromosome inactivation. Development.

[b114] King M (1993). Species evolution: the role of chromosome change.

[b115] Crow TJ (2000). Schizophrenia as the price that Homo sapiens pays for language: a resolution of the central paradox in the origin of the species. Brain Res. Rev.

[b116] Crow T (2012). Schizophrenia as variation in the sapiens-specific epigenetic instruction to the embryo. Clin. Genet.

